# Clinical, virological and biochemical evidence supporting the association of HIV-1 reverse transcriptase polymorphism R284K and thymidine analogue resistance mutations M41L, L210W and T215Y in patients failing tenofovir/emtricitabine therapy

**DOI:** 10.1186/1742-4690-9-68

**Published:** 2012-08-13

**Authors:** Gilberto Betancor, César Garriga, Maria C Puertas, María Nevot, Lourdes Anta, José L Blanco, M Jesús Pérez-Elías, Carmen de Mendoza, Miguel A Martínez, Javier Martinez-Picado, Luis Menéndez-Arias

**Affiliations:** 1 Centro de Biología Molecular “Severo Ochoa”, Consejo Superior de Investigaciones Científicas & Universidad Autónoma de Madrid, Madrid, Spain; 2 Centro Nacional de Epidemiología, Instituto de Salud Carlos III, Madrid, Spain; 3 AIDS Research Institute IrsiCaixa, Institut d’Investigació en Ciències de la Salut Germans Trias i Pujol, Universidad Autònoma de Barcelona, Badalona, Spain; 4 Hospital Carlos III, Madrid, Spain; 5 Hospital Clìnic, Barcelona, Spain; 6 Hospital Ramón y Cajal (Instituto Ramón y Cajal de Investigación Sanitaria), Madrid, Spain; 7 Institució Catalana de Recerca i Estudis Avançats, Barcelona, Spain

## Abstract

**Background:**

Thymidine analogue resistance mutations (TAMs) selected under treatment with nucleoside analogues generate two distinct genotypic profiles in the HIV-1 reverse transcriptase (RT): (i) TAM1: M41L, L210W and T215Y, and (ii) TAM2: D67N, K70R and K219E/Q, and sometimes T215F. Secondary mutations, including thumb subdomain polymorphisms (*e.g.* R284K) have been identified in association with TAMs. We have identified mutational clusters associated with virological failure during salvage therapy with tenofovir/emtricitabine-based regimens. In this context, we have studied the role of R284K as a secondary mutation associated with mutations of the TAM1 complex.

**Results:**

The cross-sectional study carried out with >200 HIV-1 genotypes showed that virological failure to tenofovir/emtricitabine was strongly associated with the presence of M184V (*P <* 10^-10^) and TAMs (*P <* 10^-3^), while K65R was relatively uncommon in previously-treated patients failing antiretroviral therapy. Clusters of mutations were identified, and among them, the TAM1 complex showed the highest correlation coefficients. Covariation of TAM1 mutations and V118I, V179I, M184V and R284K was observed. Virological studies showed that the combination of R284K with TAM1 mutations confers a fitness advantage in the presence of zidovudine or tenofovir. Studies with recombinant HIV-1 RTs showed that when associated with TAM1 mutations, R284K had a minimal impact on zidovudine or tenofovir inhibition, and in their ability to excise the inhibitors from blocked DNA primers. However, the mutant RT M41L/L210W/T215Y/R284K showed an increased catalytic rate for nucleotide incorporation and a higher RNase H activity in comparison with WT and mutant M41L/L210W/T215Y RTs. These effects were consistent with its enhanced chain-terminated primer rescue on DNA/DNA template-primers, but not on RNA/DNA complexes, and can explain the higher fitness of HIV-1 having TAM1/R284K mutations.

**Conclusions:**

Our study shows the association of R284K and TAM1 mutations in individuals failing therapy with tenofovir/emtricitabine, and unveils a novel mechanism by which secondary mutations are selected in the context of drug-resistance mutations.

## Background

Highly active antiretroviral therapy (HAART) regimens containing two nucleoside reverse transcriptase inhibitors (NRTIs) and either a non-nucleoside RT inhibitor (NNRTI) or a ritonavir-boosted protease inhibitor have become standard practice in the treatment of human immunodeficiency virus type 1 (HIV-1) infection. Despite the efficacy of current HAART regimens, the emergence of drug resistance is still a major threat to therapy success [1]. Reverse transcriptase (RT) inhibitors have been prescribed since the approval of zidovudine (AZT, 3´-azido-3´-deoxythymidine) in 1987, and NRTIs have been extensively used for the last 25 years [2,3]. Therefore, the burden of drug resistance among previously treated patients poses additional risk of therapy failure in those individuals.

NRTIs mimic natural nucleosides that are converted to triphosphate derivatives inside the cell. In this form, NRTIs act as competitive inhibitors of HIV-1 RT. NRTIs lack a 3´-OH group in their ribose ring, and their incorporation into the newly synthesized DNA results in chain termination [2,4]. Mutations conferring resistance to NRTIs can act by improving discrimination against nucleotide analogues [5-7] or by enhancing the excision of the inhibitor from the terminated DNA chain through phosphorolysis mediated by ATP or pyrophosphate (PPi) [8,9]. Mutations such as M184V or M184I conferring resistance to lamivudine or emtricitabine are known to affect nucleotide discrimination [5]. However, combinations of M41L, D67N, K70R, L210W, T215F/Y and K219E/Q increase ATP-mediated excision of chain-terminating NRTIs (reviewed in ref. [4]). DNA primers terminated with thymidine analogues (AZT or 2´,3´-didehydro-2´,3´-dideoxythymidine (d4T, stavudine)) or tenofovir are good substrates of the excision reaction. In contrast, cytidine analogues (*e.g.* lamivudine or emtricitabine) are removed very inefficiently [9-15]; (reviewed in ref. [1]).

Sequence analysis of HIV-1 isolates from patients receiving long-term therapy with AZT and/or d4T revealed that thymidine analogue resistance mutations (TAMs) acting through the excision mechanism associated in two different clusters: TAM1 (M41L, L210W and T215Y) and TAM2 (D67N, K70R, K219E/Q, and sometimes T215F) [16-18]. In addition, deletions affecting Asp^67^ are usually linked to TAM2 mutations [19], while dipeptide insertions at positions 69–70 associate with both TAM1 and TAM2 clusters [20]. TAM1 is more prevalent and confers a higher degree of resistance to thymidine analogues. The factors determining the selection of TAM1 or TAM2 pathways are not known, although the sequence background of the viral population could have a significant influence.

Standard genotypic analysis is usually restricted to HIV-1 RT residues 1 to 240, including the fingers and palm subdomains of the viral polymerase. Large cross-sectional studies have shown an association between TAMs and mutations at codons 35, 39, 43, 122, 203, 207, 208, 214, 218, 223 and 228 of the RT-coding region in HIV-1 isolates from patients failing NRTI-based therapy [21-23]. Studies with recombinant HIV-1 have shown that the amino acid substitutions K43E, Q207D and F214L influence the viral replication capacity in the presence of TAMs [24-26]. More recently, several studies have demonstrated that RT residues in the thumb-connection subdomains (residues 241 to 424) and in the RNase H domain (425 to 560) could modulate NRTI susceptibility [27-30]. Examples are E312Q, G335C/D, N348I, A360I/V, V365I and A376S in the HIV-1 RT connection subdomain, and Q509L, H539N and D549N in the RNase H domain [29-31]. In the presence of TAMs, those amino acid substitutions seem to promote AZT resistance by altering the balance between ATP-dependent excision of AZT-terminated primers and RNase H degradation, on RNA/DNA template-primers [32-34]. However, there are also mutations that enhance the effect of TAMs through an RNase H-independent mechanism, either by increasing the RT processivity (*e.g.* N348I or A360V) [34] or its affinity for short RNA/DNA duplexes (*e.g.* Q509L) [32].

Previously, we showed that RT thumb subdomain polymorphisms associated with nucleoside analogue therapy failure (*i.e.* P272A, R277K and T286A) altered the HIV-1 replication capacity in the presence and in the absence of TAMs, such as M41L and T215Y [35,36]. RTs with the combination Pro^272^/Arg^277^/Thr^286^ showed increased efficiency in rescuing DNA primers terminated with thymidine analogues and annealed to RNA templates [36]. Those effects were related to the higher affinity for RNA/DNA complexes shown by RTs having Pro, Arg, and Thr at positions 272, 277 and 286, respectively, but were independent of their intrinsic RNase H activities. Genotypic analyses carried out with large databases including treated and naïve HIV-infected patients from the U.S. and Europe showed the significantly higher prevalence of R284K in the treated population [37] and its association with the accumulation of TAMs [22]. According to the Stanford HIV Drug Resistance Database (http://hivdb.stanford.edu/; accessed on July 6^th^, 2012), the frequency of R284K in HIV-1 group M – subtype B RTs found in naïve patients is estimated at 1.9%, while this figure goes up to 4.1% when the analysis is carried out with sequences from NRTI-treated individuals.

In this work, we show that R284K is associated with TAM1 mutations in patients failing treatment with tenofovir/emtricitabine-based therapies. The significance of this association is supported by the results of viral replication assays carried out in the presence of AZT and tenofovir. Enzymological studies provide evidence on the role of R284K as a polymorphism that enhances primer rescue by promoting DNA synthesis after NRTI excision.

## Results

### Mutations associated with tenofovir/emtricitabine-based therapy failure

After approval of tenofovir disoproxil fumarate and emtricitabine, this combination has been frequently used in rescue therapies with patients failing HAART regimens. However, the presence of drug-resistance mutations at baseline, particularly TAMs, limits the efficacy of such a treatment. We have performed a retrospective analysis of RT sequences obtained from patients treated in Spanish hospitals that failed combination therapies including tenofovir and emtricitabine. A total of 222 HIV-1 genotypes were examined, 118 of them were from naïve individuals and 104 from previously-treated patients that did not respond to therapies including tenofovir and emtricitabine as the only nucleoside analogues in the salvage regimen. In most cases, salvage therapies involved co-administration of NRTIs together with protease inhibitors (64.4% of the patients), NNRTIs (usually efavirenz) (26.9% of the patients) or with both groups of drugs. All therapies included at least three different drugs.

The comparison of RT mutation frequencies in treated and naïve populations revealed that M184V was strongly associated with virological failure (viral load above 1000 RNA copies/ml) (*P* < 2 x 10^-14^) (Table 1), although several TAMs (*e.g.* M41L, D67N, L210W, T215F, etc.…) and NNRTI resistance mutations (e.g. K103N, Y181C and G190A) were also associated with therapy failure, all with *P* values below 5 x 10^-4^. The frequency of accessory mutations such as T39A, H208Y and L228H was also significantly higher in the treated population compared with the naïve group. In the RT thumb subdomain, significant differences were found for R284K and V292I whose frequency was 3.8 and 1.9 times higher in the population failing HAART (*P* < 0.05).

**Table 1:** Nucleoside RT inhibitor-related mutation frequencies in treatment-experienced and naïve patients

	**Naïve**	**Tenofovir/Emtricitabine therapy failure**	
**Mutation**	**Mutation frequency(%)**	**Mutation frequency(%)**	***P***
**V35I**	**11.0**	**23.1**	**1.82 x 10**^**-2**^
**T39A**	**5.1**	**17.3**	**3.75 x 10**^**-3**^
**M41L**	**2.5**	**23.1**	**3.07 x 10**^**-6**^
**A62V**	**―**	**6.7**	**4.87 x 10**^**-3**^
**K65R**	**―**	**3.8**	**ns**
**D67N**	**―**	**21.2**	**1.42 x 10**^**-7**^
**T69N**	**―**	**7.7**	**2.13 x 10**^**-3**^
**K70R**	**―**	**14.4**	**2.02 x 10**^**-5**^
**L74I**	**―**	**5.8**	**1.08 x 10**^**-2**^
**A98G**	**1.7**	**7.7**	**3.65 x 10**^**-2**^
**K103N**	**4.2**	**21.2**	**1.25 x 10**^**-4**^
**V108I**	**―**	**13.5**	**3.99 x 10**^**-5**^
**V118I**	**2.5**	**13.5**	**2.46 x 10**^**-3**^
**S162A**	**8.5**	**1.9**	**3.59 x 10**^**-2**^
**Q174R**	**―**	**5.8**	**1.09 x 10**^**-2**^
**V179I**	**2.5**	**10.6**	**1.57 x 10**^**-2**^
**Y181C**	**―**	**17.3**	**2.47 x 10**^**-6**^
**M184V**	**0.8**	**42.3**	**1.76 x 10**^**-14**^
**G190A**	**―**	**15.4**	**1.00 x 10**^**-5**^
**H208Y**	**―**	**9.6**	**5.99 x 10**^**-4**^
**L210W**	**0.8**	**18.3**	**6.23 x 10**^**-6**^
**L214F**	**79.7**	**91.3**	**1.65 x 10**^**-2**^
**T215F**	**―**	**10.6**	**3.05 x 10**^**-4**^
**T215Y**	**―**	**19.2**	**6.00 x 10**^**-7**^
**K219E**	**―**	**7.7**	**2.14 x 10**^**-3**^
**K219Q**	**0.8**	**9.6**	**2.89 x 10**^**-3**^
**K223E**	**―**	**4.8**	**2.44 x 10**^**-2**^
**L228H**	**―**	**9.6**	**6.02 x 10**^**-4**^
**L228R**	**―**	**7.7**	**2.16 x 10**^**-3**^
**R284K**	**2.5**	**9.6**	**2.87 x 10**^**-2**^
**V292I**	**11.0**	**21.2**	**4.49 x 10**^**-2**^
**E297K**	**19.5**	**35.6**	**7.82 x 10**^**-3**^
**G333E**	**6.8**	**15.4**	**4.60 x 10**^**-2**^

Reported *P*-values indicate statistically significant differences, corrected for the multiple-hypothesis testing by the Benjamini-Hochberg method, with a false discovery rate of 0.05 with respect to the results from isolates from drug-naïve patients (N = 118). For the chi-square contingency tests, the therapy failure group (N = 104) included samples from patients who were receiving the inhibitor at the time of genotypic testing and showed a viral load above 1000 RNA copies per ml. ns, not significant.

### Covariation and mutational clusters

Pair-wise binary (phi) correlation coefficients were calculated to identify patterns of drug resistance mutations in patients failing treatment with tenofovir and emtricitabine. The strongest correlations between mutations associated with therapy failure were found with TAMs M41L, L210W and T215Y, which form the TAM1 cluster (Additional file 1: Table S1). Higher levels of significance were also detected for other pairs of TAMs, such as K70R and K219Q, D67N and K70R, and T215F and K219Q. Interestingly, T69N appeared to be strongly correlated with K219Q (phi = 0.76, *P* = < 10^-14^) but also with K70R (phi = 0.50, *P* < 10^-6^). M41L and T215Y were also strongly correlated with V118I (P < 10^-6^), and with accessory mutations Q174R and L228H.

**Figure 1:**
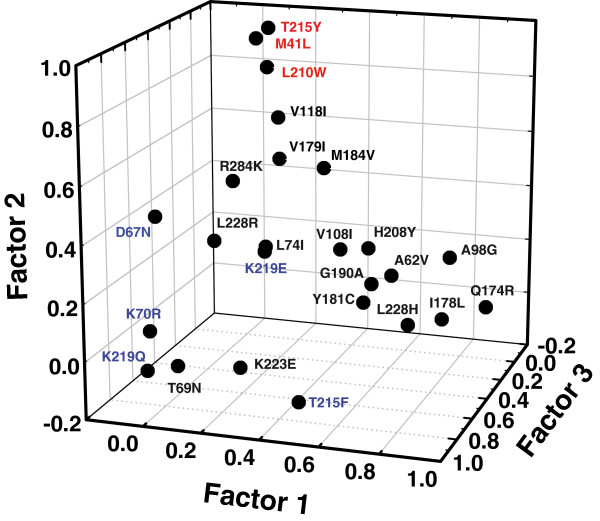
**Principal axis factoring analysis of correlations between mutations associated with tenofovir/emtricitabine therapy failure.** A factor scores plot (in rotated factor space) is shown. Amino acid changes with high coefficients of covariation are close together, while large distances separate those substitutions that show low or negative coefficients of association. Major mutations of the TAM1 and TAM2 complexes are indicated in red and blue, respectively.

Those data suggested the existence of clusters of mutations at baseline that compromised response to therapy. To identify those clusters we performed a principal axis factoring analysis. This computational procedure allows for the identification of underlying relationships between correlated mutations. The amino acid substitutions that were significant for pair-wise correlations were used in the factor analysis. The measure of sample adequacy (Kaiser-Meyer-Olkin criterion) was 0.72, and a Bartlett test of sphericity became significant (χ^2^ 1794, df = 300, p < 0.0001). Five relevant factors were obtained after using a scree plot, and taking eigenvalues greater than 1.50. These five factors explained 59.0% of the total variance of inter-correlations, while the first three explained 45.0% of the total variance (Additional file 1: Table S2). The graphical representation of these three major factors revealed three clusters of mutations: (1) A98G, Q174R, I178L and L228H (factor 1); (2) M41L, L210W and T215Y (*i.e.* TAM1 pathway) (factor 2); and (3) D67N, K70R, T215F and K219Q (*i.e.* TAM2 pathway) (factor 3) (Figure 1).

Clusters (2) and (3) include all major TAMs. No clustering was observed for the characteristic tenofovir resistance mutation K65R. Our analysis showed that mutations A62V, Y181C, G190A, H208Y and T215F could cluster with the first group, although the association was weaker than for A98G, Q174R, I178L and L228H. Amino acid substitutions D67N, L74I, K223E and L228H associate with the TAM2 cluster, while V118I, V179I, M184V and R284K are linked to cluster (2) formed by mutations of the TAM1 pathway. Among those four mutations, R284K was the only one that showed a positive value (0.356) associated with factor 2, while having negative values for all other factors included in the analysis (Additional file 1: Table S2). R284K was often identified in sequences containing one or more mutations of the TAM1 cluster, but lacking M184V. Furthermore, the strongest correlation involving R284K was found with T215Y (phi = 0.42; *P* < 2 x 10^-5^) (Additional file 1: Table S1). The association of R284K with TAM1 mutations was consistent with previous reports demonstrating the higher prevalence of R284K in the NRTI-treated population [22,37,38].

### NRTI susceptibility and replication capacity of HIV-1 with RT mutations M41L, L210W, T215Y and R284K

The association between R284K and mutations of the TAM1 cluster was tested in the presence of drugs using recombinant HIV-1. As expected, phenotypic drug susceptibility assays showed that the combination M41L/L210W/T215Y had a significant impact on AZT resistance, but a minor effect on HIV-1 susceptibility to d4T, tenofovir and emtricitabine (Table 2). Addition of R284K to the TAM1 cluster produced a subtle increase in the IC_50_ for AZT, which was 5.8 times higher than the IC_50_ obtained with the WT virus. All tested viruses were found to be susceptible to d4T and tenofovir due to the relatively high dNTP concentrations present in MT-4 cells used in these assays. Under these conditions, phosphorolysis depending on ATP or PPi might be inhibited due to the formation of “dead-end complexes” [9,39], and therefore the contribution of M41L, L210W and T215Y to tenofovir and d4T resistance was barely detectable.

**Table 2:** Susceptibility of HIV-1 constructs to nucleoside RT inhibitors

	**IC**_**50**_**(nM)**			
**RTs**	**AZT**	**d4T**	**Tenofovir**^**a**^	**Emtricitabine**
WT	2.3 ± 1.5	276.0 ± 90.5	6.7 ± 2.2	144.3 ± 69.9
R284K	1.7 ± 1.1 (0.7)	130.0 ± 24.8 (0.5)	12.1 ± 5.3 (1.8)	104.2 ± 81.5 (0.7)
M41L/L210W/T215Y	11.7 ± 1.1 (5.1)	217.0 ± 75.5 (0.8)	11.0 ± 4.6 (1.6)	226.9 ± 113.2 (1.6)
M41L/L210W/T215Y/R284K	13.3 ± 1.5 (5.8)	287.3 ± 84.5 (1.0)	18.1 ± 4.4 (2.7)	294.7 ± 93.7 (2.0)

The IC_50_ values represent averages ± standard deviations of at least three tests, with each one performed six times. The fold increase in IC_50_ relative to the wild-type HXB2 virus control carrying the RT sequence of BH10 is shown between parentheses.

^a^ Experiments were carried out with the water soluble diester prodrug tenofovir disoproxil fumarate.

**Figure 2:**
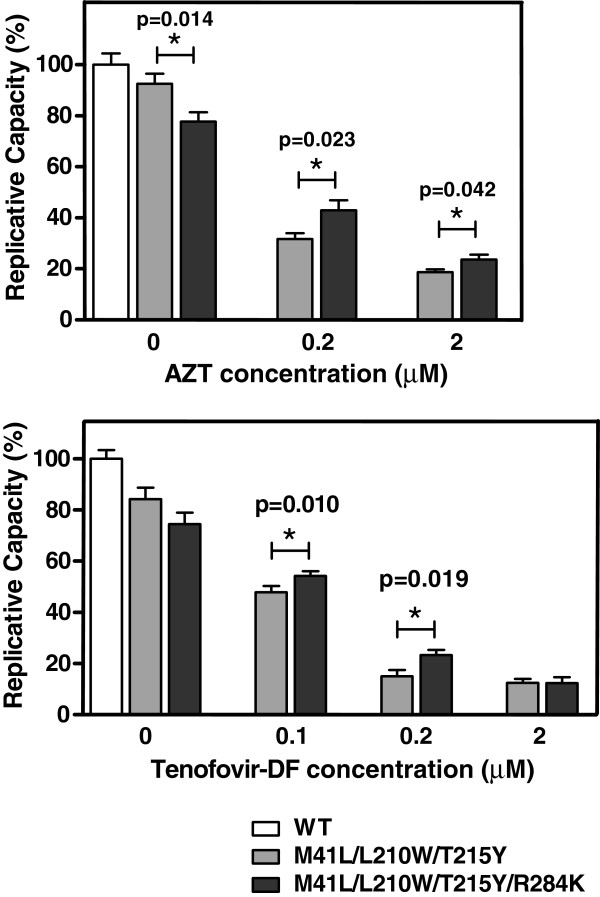
**Replication kinetics of WT and mutant RTs in the absence and presence of AZT and tenofovir disoproxil fumarate (Tenofovir-DF).** In each case, histograms show the relative replication capacity (%), compared to the WT virus in the absence of drug, based on the slopes of p24 antigen production of each recombinant virus after infection of stimulated PBMCs. The significance of the difference between slopes was calculated using the GraphPrism v. 4 software and significant *p* values are represented above the bars. Statistical analyses were performed by using a Student *t* test.

In the presence of tenofovir, growth competition assays carried out in MT-4 cells failed to show significant differences between HIV-1 clones containing RT variants M41L/L210W/T215Y and M41L/L210W/T215Y/R284K (data not shown). Therefore, viral fitness was assayed in peripheral blood mononuclear cells (PBMCs), in the presence of AZT and tenofovir. The lower nucleotide concentrations found in primary human PBMCs facilitate the detection of minor differences in virus replication efficiencies [40]. Mutants M41L/L210W/T215Y and M41L/L210W/T215Y/R284K grown in PBMCs in the absence of drugs showed decreased replication capacity compared to the WT virus (Figure 2). Under these conditions, R284K had a detrimental effect on viral fitness. However, in the presence of AZT or tenofovir, this amino acid change produced a significant increase in the replication capacity of HIV-1 bearing mutations M41L, L210W and T215Y. Thus, R284K had a compensatory effect on the loss of fitness caused by the TAM1 cluster of mutations, in the presence of NRTIs.

### Contribution of R284K to ATP-mediated rescue of primers terminated with NRTIs

**Figure 3:**
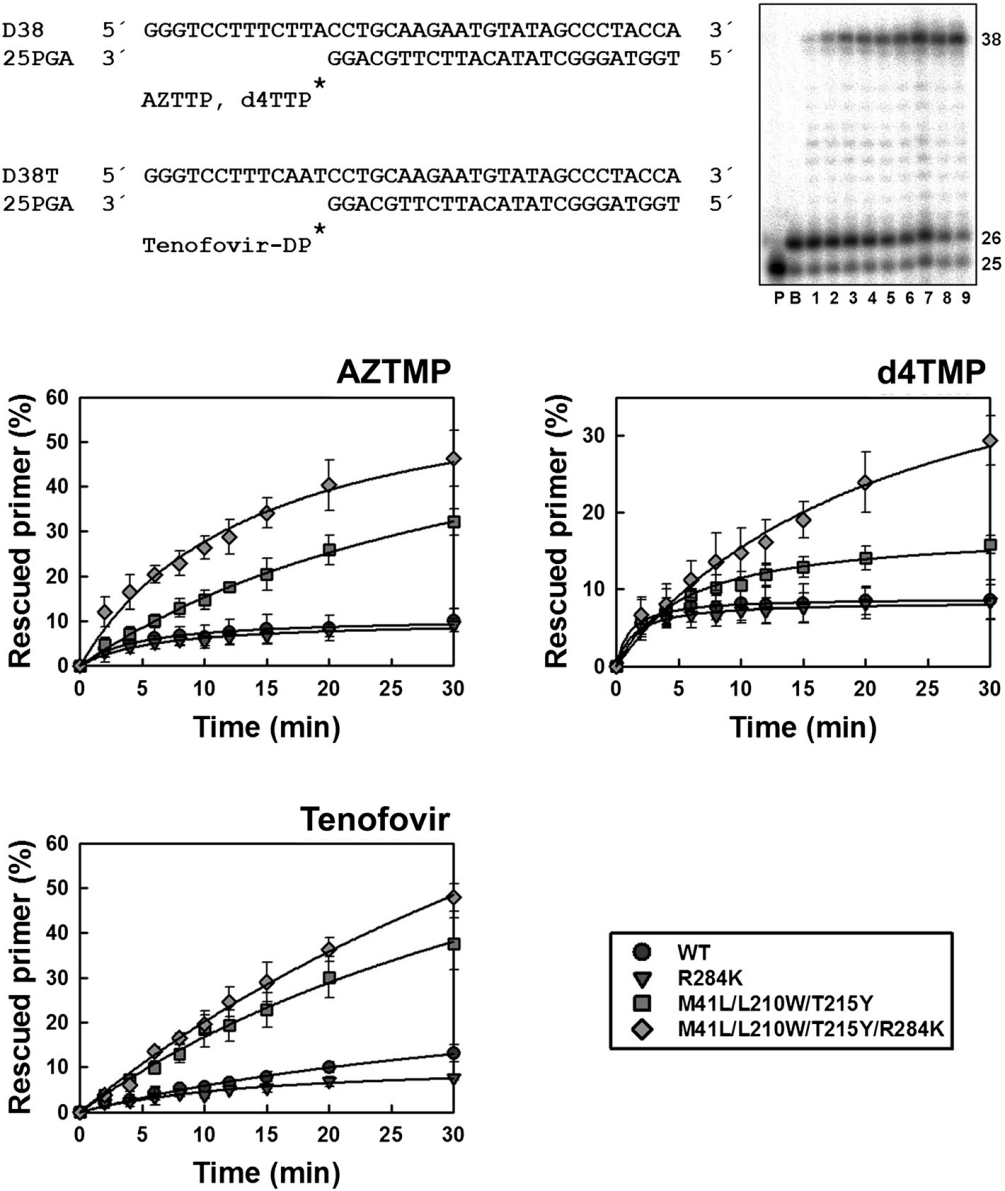
**Rescue DNA polymerization initiated from AZTMP-, d4TMP-, and tenofovir-terminated primers annealed to a DNA template.** Reactions were carried out with D38/25PGA or D38T/25PGA complexes (sequences given above). The 25-nucleotide primer (lane P) is first blocked with the nucleotide analogue (lane B). The excision of the inhibitor, followed by extension of the primer is achieved after addition of a mixture containing 3.2 mM ATP and the four dNTPs. A fully extended 38-nucleotide product is formed. The gel on the right shows a representative time course experiment of a primer rescue reaction. Lanes 1 to 9 correspond to aliquots removed 2, 4, 6, 8, 10, 12, 15, 20, and 30 minutes after the addition of 3.2 mM ATP. Graphs of time course experiments of primer rescue reactions initiated from inhibitor-terminated primers are given below. All dNTPs in the assays were supplied at 100 μM, except for dATP or dTTP (depending on the reaction) whose concentration was 1 μM. Template-primer and active RT concentrations in these assays were 30 and 24 nM, respectively. The values (averaged ± standard deviations [error bars]) were obtained from three independent experiments.

The ability of RTs to unblock AZTMP-, d4TMP- and tenofovir-terminated primers was first assessed with DNA/DNA template-primers (Figure 3). In the presence of 3.2 mM ATP, we observed that with all NRTIs, the addition of R284K to a mutational background constituted by M41L, L210W and T215Y produced a significant increase in the amount of rescued and fully extended primers. These effects were more pronounced for primers terminated with thymidine analogues than for those blocked with tenofovir, but differences were significant in all cases. The R284K alone had no significant effect on the ATP-mediated rescuing ability of the WT RT.

**Figure 4:**
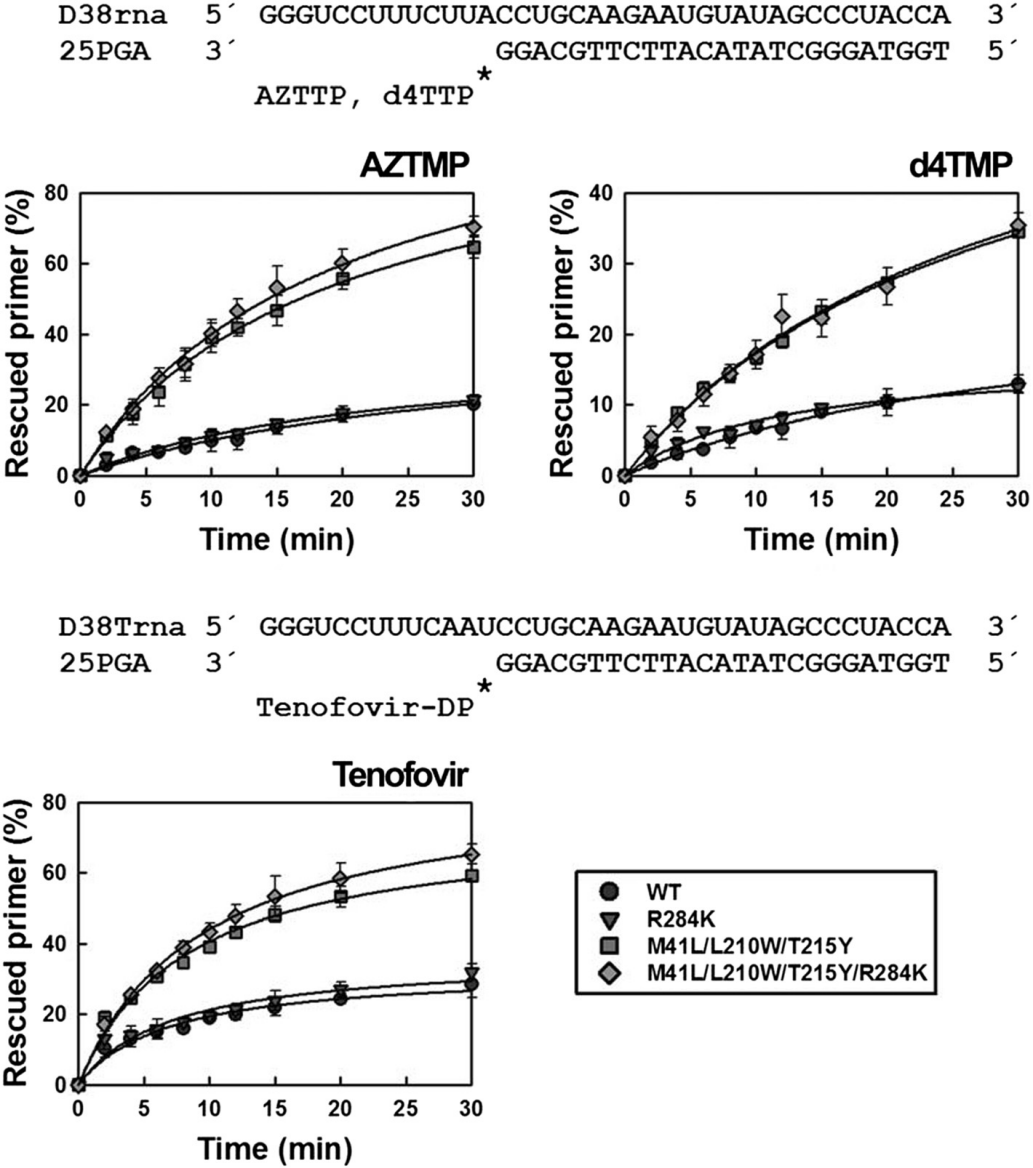
**Rescue DNA polymerization initiated from AZTMP-, d4TMP-, and tenofovir-terminated primers annealed to an RNA template.** Time course experiments of excision reactions were carried out in the presence of 3.2 mM ATP. The nucleotide sequences of template-primers used are given above their corresponding graphs. All dNTPs in the assays were supplied at 200 μM, except for dATP or dTTP (depending on the reaction) whose concentration was 2 μM. Template-primer and active RT concentrations in these assays were 30 and 24 nM, respectively. The values (averages standard deviations [error bars]) were obtained from three independent experiments.

**Figure 5:**
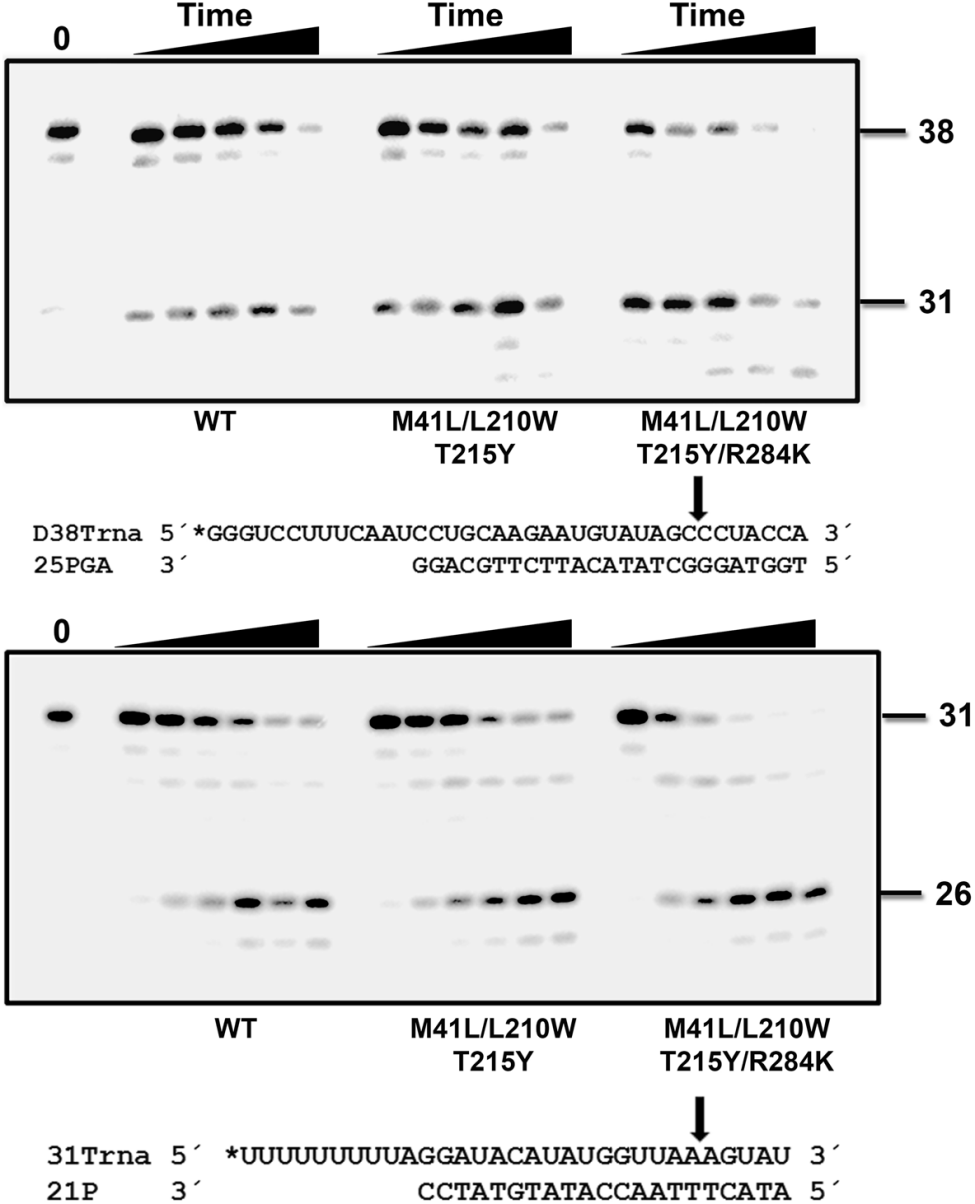
**RNase H activity of wild-type and mutants RTs M41L/L210W/T215Y and M41L/L210W/T215Y/R284K.** [^32^P]RNA/DNA substrates (50 nM) were cleaved at 37°C in the presence of the corresponding RT at 50 nM concentration. Template-primer sequences are shown below. Arrows in the template sequences indicate the cleavage sites. For D38Trna/25PGA, the time points were taken after incubating the samples for 20 s, 40 s, and 1, 2 and 4 minutes. Catalytic rate constants for the cleavage of D38Trna were 0.34 ± 0.15 min^-1^, 0.39 ± 0.18 min^-1^ and 1.16 ± 0.54 min^-1^ for WT, and mutant RTs M41L/L210W/T215Y and M41L/L210W/T215Y/R284K, respectively. For 31Trna/21P, the time points were drawn after 20 s, 40 s, and 1, 2, 3 and 4 minutes. The catalytic rate constants with this substrate were 0.33 ± 0.05 min^-1^ for WT RT, and 0.35 ± 0.03 min^-1^ and 0.87 ± 0.12 min^-1^ for mutants M41L/L210W/T215Y and M41L/L210W/T215Y/R284K, respectively. Kinetic data were averages of three independent experiments.

The differences found using DNA/DNA complexes were not observed with RNA/DNA template-primers (Figure 4). Although mutant RTs M41L/L210W/T215Y and M41L/L210W/T215Y/R284K showed remarkable ATP-dependent phosphorolytic activity, the presence of R284K had a minimal impact on rescue DNA polymerization initiated from NRTI-terminated primers. RNase H activity assays carried out with the RNA/DNA template-primer used in ATP-mediated rescue experiments demonstrated that the M41L/L210W/T215Y/R284K RT had increased endonucleolytic activity in comparison with M41L/L210W/T215Y RT (Figure 5).

The reduced stability of the RNA template in reactions catalyzed by M41L/L210W/T215Y/R284K RT is consistent with a reduction in the elongation efficiency of unblocked DNA primers in rescue assays. The mutant M41L/L210W/T215Y/R284K RT also showed increased RNase H activity in comparison with the WT enzyme, as demonstrated in assays carried out with RNA/DNA duplexes 38Trna/25PGA and 31Trna/21P (Figure 5). For example, in assays carried out with 31Trna/21P, the amount of RNA substrate remaining after 3 minutes of incubation was <8% for the M41L/L210W/T215Y/R284K mutant RT, and 35% for WT and M41L/L210W/T215Y RTs. The catalytic rate constants for RNA template cleavage obtained with the M41L/L210W/T215Y/R284K RT were around 2.5-3 times higher than those obtained with M41L/L210W/T215Y and WT RTs. An increased frequency of RNase H secondary cleavages was also observed with 38Trna/25PGA in reactions catalyzed by the M41L/L210W/T215Y/R284K RT. However, catalytic rates for these secondary cleavages could not be determined accurately.

**Figure 6:**
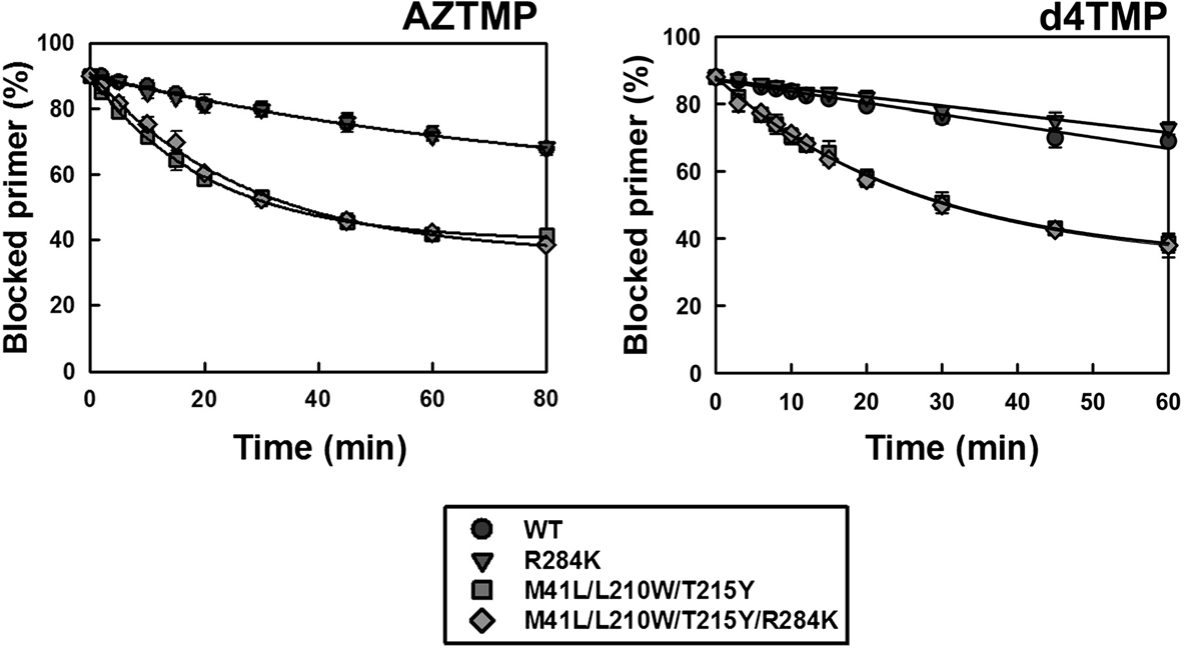
**Kinetics of the ATP-dependent excision of AZTMP and d4TMP from DNA/DNA template-primers.** Time course experiments for the excision reaction of AZTMP- and d4TMP-terminated primers (26-mers) annealed to their corresponding 38-nucleotide DNA templates (30 nM) were determined in the presence of 3.2 mM ATP. The excision reaction was catalyzed by WT and mutant RTs (210 nM). The calculated *k*_obs_ values for the AZTMP excision reaction were 2.82 x 10^-3^ ± 1.48 x10^-4^ min^-1^ for WT RT, 2.42 x 10^-3^ ± 1.22 x 10^-4^ min^-1^ for mutant R284K RT, 4.69 x 10^-2^ ± 1.69 x 10^-3^ min^-1^ for M41L/L210W/T215Y RT, and 3.64 x10^-2^ ± 3.08 x 10^-3^ min^-1^ for M41L/L210W/T215Y/R284K RT. For the excision of d4TMP, the *k*_*obs*_ values for WT and mutants R284K, M41L/L210W/T215Y, and M41L/L210W/T215Y/R284K were 3.37 x 10^-3^ ± 1.93 x 10^-4^ min^-1^, 2.62 x 10^-3^ ± 1.42 x 10^-4^ min^-1^, 3.72 x 10^-2^ ± 1.58 x 10^-3^ min^-1^, and 3.70 x 10^-2^ ± 2.29 x 10^-3^ min^-1^, respectively.

Rescue DNA polymerization assays involve the excision of the NRTI and the subsequent extension of the unblocked primer. Excision rates for mutant and WT RTs were obtained under single-turnover conditions with primers blocked with thymidine analogues, and annealed to a DNA template (Figure 6). TAM-containing RTs showed similar excision rates for AZTMP- and d4TMP-terminated primers, and ranged from 0.036 to 0.047 min^-1^. The presence of R284K did not affect ATP-mediated excision of any thymidine analogue. On the other hand, as expected, WT and mutant R284K RTs showed much lower excision rates (<0.0034 min^-1^).

### R284K increases the DNA polymerization rate when combined with M41L, L210W and T215Y

The results described above suggested that rescue efficiency differences between M41L/L210W/T215Y and M41L/L210W/T215Y/R284K RTs could originate from an altered DNA binding affinity. However, the equilibrium dissociation constants (*K*_d_) for WT and mutant RTs and DNA/DNA template-primers showed that differences between enzymes were not significant (Additional file 1: Table S3). M41L/L210W/T215Y/R284K RT showed a slightly higher processivity on heteropolymeric DNA substrates in comparison with the M41L/L210W/T215Y RT (Additional file 1: Figure S2). However, the dissociation rate constants (*k*_off_) for DNA/DNA template-primers were similar for both enzymes (*i.e.* 0.215 ± 0.030 s^-1^ for M41L/L210W/T215Y RT and 0.201 ± 0.033 s^-1^ for M41L/L210W/T215Y/R284K RT).

Interestingly, M41L/L210W/T215Y/R284K RT showed increased elongation rates compared with the M41L/L210W/T215Y RT in assays carried out with unblocked primers (Additional file 1: Figure S1). These differences were observed at different concentrations of DNA/DNA template-primer and in the presence of relatively high concentrations of dNTP. Additional experiments carried out with RTs lacking ATP-dependent excision activity (*i.e*. WT and R284K RTs) also showed that the 284 mutation increased primer elongation, particularly when the concentrations of template-primer were not limiting (Additional file 1: Figure S1).

Steady-state kinetic parameters for dTTP and dATP incorporation showed that M41L/L210W/T215Y/R284K RT had an increased catalytic rate of nucleotide incorporation (*k*_cat_), in comparison with mutant M41L/L210W/T215Y and the WT enzyme (Table 3). In the case of dATP incorporation, the catalytic efficiency (*k*_cat_/*K*_m_) of M41L/L210W/T215Y/R284K RT was also 2.6 times higher than the catalytic efficiency of the mutant M41L/L210W/T215Y. Taken together, those data suggest that R284K improves the rescue efficiency of M41L/L210W/T215Y RT by promoting dNTP incorporation, particularly in the presence of relatively high concentrations of nucleotide. On the other hand, R284K appears to have a minimal impact on AZTTP and tenofovir-DP binding, since the inhibition constants (*K*_i_) obtained with WT and mutant RTs under our assay conditions were similar and around 2 to 3 μM (Table 3).

**Table 3:** Steady-state kinetic parameters of nucleotide incorporation catalyzed by wild-type and mutant RTs, and inhibition constants for AZTTP and tenofovir-DP

**RTs**	**Nucleotide**	***k***_**cat**_**(min**^**-1**^**)**	***K***_**m**_**(nM)**	***k***_**cat**_**/*K***_**m**_**(μM**^**-1**^** min**^**-1**^**)**	***K***_**i**_**(AZTTP) (μM)**	***K***_**i**_**(Tenofovir-DP) (μM)**
WT	dTTP	3.14 ± 0.55	108.3 ± 12.9	30.9 ± 4.7	2.10 ± 0.38	
	dATP	3.87 ± 0.99	25.8 ± 6.7	172.0 ± 27.7		3.59 ± 0.26
M41L/L210W/T215Y	dTTP	3.53 ± 0.09	117.4 ± 21.9	30.8 ± 6.0	2.18 ± 0.17	
	dATP	5.70 ± 0.87	32.1 ± 9.6	243.9 ± 51.1		2.63 ± 0.64
M41L/L210W/T215Y/R284K	dTTP	5.66 ± 0.23	184.5 ± 6.1	30.7 ± 2.3	2.32 ± 0.74	
	dATP	10.09 ± 1.47	15.9 ± 2.5	639.6 ± 101.2		2.54 ± 0.55

D38/25PGA and D38T/25PGA were used as substrates for dTTP and dATP incorporation, respectively. Reported values are the averages ± standard deviations, obtained from at least three independent experiments.

It should be noted that although steady-state rates (*k*_cat_) are dominated by the slowest step of the polymerization reaction (*i.e*. dissociation of the RT-DNA complex) [41], the observed *k*_cat_ differences between M41L/L210W/T215Y and M41L/L210W/T215Y/R284K RTs are expected to be relevant because both enzymes have similar *k*_off_ values. Further support of this proposal was obtained from single-nucleotide incorporation assays carried out under pre-steady-state conditions, with template-primer D38/25PGA and at a high dTTP concentration (*i.e*. 80 μM). In these experiments, M41L/L210W/T215Y and M41L/L210W/T215Y/R284K RTs showed nucleotide incorporation rates (*k*_obs_) of 21.5 ± 4.4 s^-1^ and 38.8 ± 9.4 s^-1^, respectively. These results were consistent with an increase in the polymerization rate mediated by R284K when introduced in the context of TAM1 mutations.

## Discussion

Previous reports showed that the presence of three or more TAMs (particularly, M41L, T215Y and/or L210W) compromised the efficacy of tenofovir [42-44]. These mutations arise after extensive or suboptimal treatment with nucleoside analogues, particularly with AZT or d4T. In agreement with those findings, our cross-sectional study involving previously treated patients reveals that the presence of TAMs and M184V contributes to failure of salvage therapies containing the combination of tenofovir and emtricitabine, while K65R is relatively rare in those patients. These results were also consistent with the low prevalence of K65R in the presence of TAMs, in patients receiving tenofovir-containing combination therapies [43,45,46]. K65R exerts an antagonistic effect on TAMs, by decreasing the ATP-mediated phosphorolytic activity that facilitates removal of NRTIs (*i.e*. thymidine analogues and tenofovir, among others) from blocked DNA primers [47]; (reviewed in ref. [4]).

Several NNRTI resistance mutations were found to be more prevalent in the group of patients failing therapy. In some cases, the presence of these mutations can be attributed to selection with efavirenz (*e.g*. K103N), since this drug had been co-administered with tenofovir/emtricitabine in 20.2% of the non-responding individuals. Otherwise, NNRTI resistance mutations may have arisen from previous treatments with NNRTIs. The preservation of NNRTI mutations in the viral genome of treated patients can be attributed to their small effect on viral fitness, as demonstrated for V108I, Y181C and G190A [48-51]. Our bivariate analysis also showed that accessory mutations at positions 35, 39, 162, 174, 179, 208, 223, 228, 284, 292, 297 and 333 could be related to therapy failure. Among them, T39A, V179I, H208Y, K223E, L228H/R, R284K and V292I have been previously identified as secondary mutations associated with the accumulation of TAMs and with resistance to nucleoside analogues [21,22,35,37,52,53]. On the other hand, L74V, V179I, K223E and L228H/R were previously identified as modulators of NNRTI resistance in a large study carried out in central Italy [23].

Our analysis clearly shows a separate clustering of TAMs in two different groups that coincides with major selection pathways, previously identified in patients treated with thymidine analogues (*i.e*. TAM1 and TAM2) [16-18]. Thus, TAM1 includes the amino acid substitutions M41L, L210W and T215Y, while TAM2 contains mutations D67N, K70R, K219E/Q and sometimes T215F. In our data set, the TAM1 pathway includes all three mutations that show high phi values in the correlation analysis. V118I, V179I, M184V and R284K appear to be associated with the TAM1 pathway. V118I and M184V are mutations selected under therapy with lamivudine (reviewed in refs. [1,4]), while V179I has been associated with resistance to NNRTIs [54].

Interestingly, the prevalence of R284K seems to increase with the exposure to nucleoside RT inhibitors. In a study involving more than 2,000 patients treated in hospitals in the U.K., Cane *et al*. [22] showed that the prevalence of R284K increased with the number of TAMs found in the viral genotype. Only one percent of the isolates lacking TAMs were found to contain the mutation. However, the prevalence of R284K increased up to 4.1%, 6.5% and 9.3% in isolates having 2, 3 or 4 TAMs, respectively. Further evidence of this association was reported by a study of the Swiss Cohort that showed that the frequency of R284K increased from 1.1% in naïve individuals to 5.1% in patients treated with thymidine analogues [38]. Interestingly, that analysis also showed that in those individuals exposed to thymidine analogues and didanosine, the frequency of R284K was 6.0%, while the frequencies of M41L, L210W, T215Y and M184V were 48.3%, 36.8%, 57.7% and 1.0%, respectively. These data together with the strong correlation of R284K and T215Y in our database suggest that the emergence of R284K is not related to the selection of the emtricitabine resistance mutation M184V.

Our results show that R284K has no effect on the viral susceptibility to thymidine analogues and tenofovir of isolates containing the TAM1 complex (*i.e.* M41L/L210W/T215Y). However, it confers a fitness advantage when the virus is grown in PBMCs in the presence of drug. Arg^284^ is located in the thumb subdomain close to the template strand and could influence ATP-dependent excision reaction in a similar way as that reported for other amino acid substitutions in the HIV-1 RT thumb subdomain (*i.e.* P272A/R277K/T286A) [36]. When combined, those three polymorphisms had a negative effect on the efficiency of rescue reactions carried out with blocked primers complexed with RNA, and these effects were due to the lower affinity of the mutant RT for RNA/DNA complexes.

Our biochemical studies showed that in the context of M41L/L210W/T215Y, the substitution of Lys for Arg^284^ has no effect on ATP-dependent excision. However, R284K increases the efficiency of the rescue reaction and facilitates the elongation of the unblocked primers. This effect cannot be attributed to an influence on nucleic acid binding or processivity but to the higher catalytic efficiency of the M41L/L210W/T215Y/R284K RT in comparison with the M41L/L210W/T215Y enzyme. In combination with TAM1 mutations, R284K produced 60-77% increase in the catalytic rate (*k*_cat_) for nucleotide incorporation. This effect was also consistent with the higher efficiencies of primer extension observed with the M41L/L210W/T215Y/R284K RT, using different concentrations of template-primer. Despite differences in the catalytic parameters, nucleotide discrimination seems to play a minor role in the effects of R284K, since AZTTP and tenofovir-DP inhibition constants (*K*_i_) were similar for both enzymes. Taken together, these results could explain why R284K confers a small fitness advantage in the presence of nucleoside analogues, as shown in the viral replication assays.

Interestingly, the differences in rescue efficiencies between M41L/L210W/T215Y/R284K and M41L/L210W/T215Y RTs were found with DNA/DNA template-primers, but not with RNA/DNA complexes. RNase H assays carried out with both mutants and the wild-type RT revealed the higher endonucleolytic activity of M41L/L210W/T215Y/R284K RT in comparison with WT and mutant M41L/L210W/T215Y RTs. Therefore, the minor differences in rescue efficiencies observed with AZTMP-, d4TMP- or tenofovir-terminated primers complexed with RNA could be explained by the poor stability of the template in rescue assays carried out with the M41L/L210W/T215Y/R284K mutant. It is also possible that the emergence of R284K in RTs having TAM1 mutations could have a negative impact on the stability of the viral RNA during reverse transcription, thereby explaining its small but significant reduction in viral fitness in the absence of inhibitors. Nevertheless, other undetermined factors may also contribute to this effect. For example, the potential effects of thumb subdomain mutations on the stability of the p66/p51 heterodimer [55,56].

The mechanism by which R284K confers a selective advantage in the context of TAM1 mutations is different from others previously described for mutations in the thumb-connection subdomains and in the RNase H domain. Thus, mutations such as N348I, A360V and Q509L increased chain-terminated primer rescue with RNA/DNA complexes, but not with DNA/DNA template-primers [29,30,32-34,57,58]. In combination with TAMs, N348I and A360V decreased the efficiency of RNase H cleavage and increased excision of AZT in the presence of ATP [34]. Other mutations, such as the complex P272A/R277K/T286A and G333D had a minor effect on RNase H activity, but increased chain-terminated primer rescue with both RNA/DNA and DNA/DNA template-primers [36,59]. It has been suggested that secondary mutations such as E40F and K43E [25] or L214F [26] that associate with amino acid substitutions of the TAM1 resistance pathway could increase viral fitness by influencing the catalytic efficiency of the RT. However, the suggested effects were not supported by biochemical evidence. In our study we provide an example of a mutation that improves viral fitness in the presence of antiretroviral drugs by increasing its RT DNA polymerase activity, while affecting the RNase H cleavage efficiency.

## Conclusions

We have shown evidence of the association of TAM1 mutations (*i.e.* M41L/L210W/T215Y) with R284K in previously-treated patients failing salvage therapy with tenofovir/emtricitabine. Virological studies reveal that the combination of R284K with TAM1 mutations confers a fitness advantage in the presence of nucleoside analogues (*i.e.* AZT and tenofovir), although it has minimal impact on phenotypic drug resistance. Biochemical studies with WT and mutant HIV-1 RTs showed that the impact of R284K on nucleotide discrimination or ATP-mediated excision of chain-terminated primers was not significant. However, in the presence of the M41L/L210W/T215Y complex, R284K increases the catalytic rate of nucleotide incorporation and the RNase H activity of the RT, and these effects promote chain-terminated primer rescue on DNA/DNA template-primers, but not on RNA/DNA complexes. Our study unveils a novel mechanism by which secondary mutations are selected in the context of drug-resistance mutations and provide further evidence for the effects of mutations outside the RT DNA polymerase region subjected to genotypic analysis during the antiretroviral treatment.

## Methods

### HIV-1 sequences and antiretroviral treatments

The Spanish AIDS Research Network Drug Resistance Database is an anonymous clinical database that contains viral genotypes (protease- and RT-coding regions) derived from 2104 HIV-1-infected patients, treated in 12 Spanish clinics and collected since October 1999 [60]. RT mutation frequencies observed in isolates from patients failing treatment with tenofovir and emtricitabine (without additional NRTIs) (104 genotypes) were compared with those obtained from the naïve population (118 genotypes). All of the sequences used in the comparison were obtained between December 2004 and April 2008, and were classified as of group M – subtype B (GenBank accession numbers JX271276 to JX271491). The therapy failure group included genotypes from patients that had been treated with the drug combination and showed a viral load of >1000 copies/ml. All of the patients failing tenofovir/emtricitabine had been previously treated with other RT inhibitors (with or without protease inhibitors).

### Statistical analysis of genotypic data

The frequencies of specific amino acid substitutions in the first 334 residues of the HIV-1 RT in naïve patients or in individuals failing treatment with RT inhibitors was compared using contingency tables and Pearson’s χ^2^ tests (or Fisher’s exact tests where appropriate). The Benjamini-Hochberg method was used to identify results that were statistically significant in the presence of multiple-hypothesis testing [35,61]. A false discovery rate of 0.05 was used to determine statistical significance.

Mutation covariation was analyzed by calculating the binomial (phi) correlation coefficient for the simultaneous presence of mutations at two positions in the same isolate. Statistically significant correlations were those with *P* ≤ 0.05 using a Bonferroni correction for the number of possible pair-wise combinations [61,62]. The relationship among amino acid substitutions found in drug-resistant isolates was further investigated by principal axis factoring [63]. The matrix of binomial correlation coefficients was used as a measure of similarity between mutations. The factorisability of the correlation matrix for exploratory factor analysis was judged by Kaiser-Meyer-Olkin criterion on the basis of the measure of sampling adequacy. A Bartlett test of sphericity was applied to test whether correlations differ significantly from zero, and the number of relevant factors was determined by reading a scree plot. After extraction, the resulting factors were orthogonally rotated using the Varimax procedure. Pattern coefficient cut-offs for the association of mutations were set at 0.6 for those showing a strong relationship, while values between 0.3 and 0.6 were taken as evidence of moderate covariation. Data analyses were performed with the SPSS software package (SPSS Inc, Chicago, Illinois, USA).

### Mutagenesis and recombinant HIV-1

Site-directed mutagenesis was carried out with the Quik-Change Site-Directed Mutagenesis kit (Stratagene) by following the manufacturer’s instructions, and using plasmids p66RTB [64] or p66RTB(LY) [36] as templates. The plasmid p66RTB contains the nucleotide sequence encoding for the 66-kDa subunit of HIV-1_BH10_ RT, and p66RTB(LY) encodes a p66 derivative with thymidine analogue resistance mutations M41L and T215Y. Mutagenic primers 5´-GCTGAGACAACATCTGTGGAGGTGGGGACTTACC-3´ and 5´-GGTAAGTCCCCACCTCCACAGATGTTGTCTCAGC-3´ were used to introduce L210W in plasmid p66RTB(LY). The mutation R284K was added with primers 5´-GTAAACTCCTTAAAGGAACCAAAGCACTAAC-3´ and 5´- GTTAGTGCTTTGGTTCCTTTAAGGAGTTTAC-3´. All introduced mutations were confirmed by DNA sequencing. Recombinant HIV-1 was obtained as previously described [65]. The full-length RT coding sequence DNA was amplified with primers IN5 (5′-AATTTTCCCATTAGTCCTATTGAAACTGTACCA-3′) and IN3 (5′-TCTATTCCATCYAAAAATAGTACTTTCCTGATTCC-3′), and the PCR products cotransfected in MT-4 cells with an RT-deleted HXB2-D clone, previously linearized with BstEII [66]. MT-4 cells were used to propagate the recombinant virus [36]. The nucleotide sequence of the RT-coding region was fully determined in order to check for reversions or undesired mutations. MT-4 cells and the deleted HXB2-D clone were obtained from the AIDS Reagent Program (Medical Research Council).

### HIV drug susceptibility, replication capacity and growth competition assays

Susceptibility to AZT, d4T and tenofovir was determined in MT-4 cells as previously described [36], using a multiplicity of infection of 0.003. Viable cells at different drug concentrations were quantified with a tetrazolium-based colorimetric method [67]. Replication kinetics of wild-type and mutant viruses were assayed by infecting peripheral blood mononuclear cells (PBMCs) (mixed from two healthy donors), previously stimulated with phytohemagglutinin and interleukin 2 [26]. In these assays, viral replication rates were obtained from the amounts of HIV-1 p24^Gag^ antigen produced during the exponential phase of viral growth, as described previously [26]. Growth competition assays in MT-4 cells were carried out as previously described [26], in the absence and in the presence of tenofovir disoproxil fumarate at concentrations ranging from 5 to 80 nM. A multiplicity of infection of 0.001 was used and competing viruses were mixed at a 50:50 ratio. RT inhibitors were obtained from the NIH AIDS Research and Reference Reagent Program.

### RT purification

RTs were expressed in *E. coli* XL1 Blue and purified as p66/p51 heterodimers by ionic exchange, followed by immobilized metal affinity chromatography on Ni^2+^-nitriloacetic acid-agarose [64,68]. The p66 subunit was obtained with a His_6_ tag at its C-terminal end to facilitate its purification by metal affinity chromatography. Purity of the enzymes was assessed by SDS-polyacrylamide gel electrophoresis and RT concentrations were determined by active site titration as described [69,70].

### Nucleotides, templates and primers

Stock solutions (100 mM) of dNTPs and ATP were obtained from GE Healthcare. AZTTP, d4TTP and tenofovir diphosphate were purchased from TriLink Biotechnologies (San Diego, CA), Sierra Bioresearch (Tucson, AZ), and Moravek Biochemicals (Brea, CA), respectively. Before use, nucleoside triphosphates were treated with inorganic pyrophosphatase (Roche) to remove traces of PPi [12]. Synthetic oligonucleotides 21P, 25PGA, 31Trna, D38, D38rna, D38T, D38Trna and ProLac110 were obtained from Life Technologies. M13mp2 single-stranded DNA template was purified as described [71]. Primers 25PGA and ProLac110 were labeled at their 5´ termini with [γ-^32^P]ATP (Perkin Elmer) and T4 polynucleotide kinase (Promega) before annealing them to their corresponding templates.

### Chain terminator excision assays

RT-catalyzed DNA rescue reactions were carried out with DNA/DNA and RNA/DNA duplexes, in 50 mM Hepes pH 7.0 buffer, containing 15 mM NaCl, 15 mM magnesium acetate, 130 mM potassium acetate, 1 mM dithiothreitol and 5% (wt/v) polyethylene glycol [20,36]. Briefly, phosphorylated template-primers (75 nM) were preincubated with the RT (60 nM) for 10 minutes at 37°C. Then, AZTTP, d4TTP or tenofovir-DP were added to a final concentration of 25 μM and incubated for another 10 minutes in the same conditions. Rescue reactions were initiated by adding a mixture of all dNTPs and ATP at final concentrations of 100 μM each and 3.2 mM, respectively. The final concentration of the next complementary dNTP (dATP or dTTP under our assay conditions) was kept at 1 μM in order to minimize its inhibitory effect on the rescue reaction, due to the formation of a “dead-end” complex [39]. Assays with RNA/DNA complexes such as D38rna/25PGA or D38Trna/25PGA were carried out as above, but the primer was blocked with triphosphorylated nucleoside analogues at 50 μM and dNTP concentrations in the extension reactions were kept at 200 μM. Rescue and extension reactions were incubated for 30 minutes at 37°C, with aliquots being removed at appropriate times. Reactions were stopped by adding an equal amount of sample loading buffer (10 mM EDTA in 90% formamide containing 3 mg/ml xylene cyanol FF and 3 mg/ml bromophenol blue). Products were resolved on a 20% polyacrylamide–8 M urea gel, and primer rescue was quantified by phosphorimaging, using a BAS1500 scanner (Fuji) and the program Tina version 2.09 (Raytest Isotopenmessgerate GmbH, Staubenhardt, Germany).

### Pre-steady-state kinetics of the ATP-dependent excision reaction

The primer 25PGA was blocked at its 3´ end with d4TTP using terminal deoxynucleotidyltransferase (Roche) [12], and excess nucleotides were eliminated by repeated passage through a mini Quick Spin column (Roche). The blocked primer was then labeled with [γ-^32^P]ATP and T4 polynucleotide kinase and annealed to the DNA template D38, in order to obtain the template-primer D38/25PGA^d4T^, used in the excision reactions. Since AZTTP is not a substrate of terminal deoxynucleotidyltransferase, we had to use WT HIV-1_BH10_ RT to block the template-primer D38/25PGA with AZT. For this purpose, we preincubated the template-primer D38/^32^P]25PGA (4.6 μM) with HIV-1_BH10_ RT (4 μM) in 65 μl of 50 mM Hepes pH 7.0 buffer, containing 15 mM NaCl, 15 mM magnesium acetate, 130 mM potassium acetate, 1 mM dithiothreitol, and 5% polyethylene glycol for 10 minutes at 37°C. Then, 35 μl of 6 mM AZTTP (diluted in the preincubation buffer) were added and incubated for another 30 minutes at 37°C to ensure that the primer was blocked completely. The RT was inactivated by heating the mixture for 10 minutes at 90°C. After slowly cooling the sample at room temperature, it was passaged several times through a mini Quick Spin column (Roche) to eliminate the free AZTTP. Finally, the D38/25PGA^AZT^ template-primer was obtained after precipitation with 98% ethanol. The excision of d4T monophosphate (d4TMP) from D38/25PGA^d4T^ was determined under single turnover conditions, as previously described [36]. The same conditions were used to study the excision of AZTMP from template-primer D38/25PGA^AZT^.

### DNA binding affinity

Dissociation equilibrium constants (*K*_d_) for RTs and DNA duplexes were obtained with the template-primer D38/25PGA by following a previously described procedure [36].

### Kinetics of nucleotide incorporation and determination of inhibition constants for AZTTP and tenofovir-DP

Nucleotide incorporation reactions under steady-state conditions were carried out as previously described [72]. However, in the preincubation buffer, active RT and template-primers D38/25PGA or D38T/25PGA were present at concentrations of 7–20 nM and 60 nM, respectively. Elongation rates were determined with dTTP concentrations ranging from 0.05 to 20 μM or with dATP concentrations ranging from 12 nM to 10 μM, using reaction volumes of 20 μl. Catalytic constants *k*_cat_ and *K*_m_ were determined after fitting the elongation data to the Michaelis–Menten equation as described [73]. Inhibition constants (*K*_i_) for AZTTP and tenofovir-DP were determined under the conditions used for obtaining the catalytic constants of dTTP and dATP incorporation, respectively. In order to distinguish between dTTP and AZTTP, elongation reactions were carried out under conditions that allowed the incorporation of two nucleotides (*i.e.* T at position +1 and A at position +2). For this purpose dATP was supplied at 1 μM in all assays. The competition between tenofovir-DP and dATP was analyzed with template-primer D38T/25PGA. Assays were carried out with variable concentrations of dATP (as indicated above) and 2 μM dTTP to facilitate further extension of the primer when the inhibitor was not incorporated at position +1 of the DNA primer. Data were analyzed by using Lineweaver-Burk plots. Nucleotide incorporation reactions under pre-steady-state conditions were carried out with template-primer D38/25PGA, as described [72].

### Primer extension

These experiments were carried out after preincubating the template-primer (D38/25PGA) at concentrations of 3, 10 and 20 nM with the corresponding RT (6 nM) in 25 μl or 100 mM Hepes pH 7.0 buffer, containing 30 mM NaCl, 30 mM magnesium acetate, 130 mM potassium acetate, 1 mM dithiothreitol, and 5% polyethylene glycol. After 10 minutes at 37°C, all four dNTPs were added to a final concentration of 100 μM, in a solution containing 130 mM potassium acetate, 1 mM dithiothreitol, and 5% polyethylene glycol. Aliquots of 4 μl were removed at appropriate times and quenched with an equal volume of sample loading buffer, before analyzing the products by denaturing polyacrylamide gel electrophoresis and phosphorimaging.

### Processivity

The processivity of wild-type and mutant RTs was assessed with a complex made of M13mp2 single-stranded DNA as template and ProLac110 (5’- GCGATTAAGTTGGGT-3’) as primer, and with the 38/25-mer D38/25PGA. RTs and template-primer (both at 60 nM concentration) were preincubated at 37°C for 10 minutes in 12 μl of 50 mM Tris–HCl pH 8.0 buffer, containing 50 mM KCl. Reactions were initiated by adding 12 μl of preincubation buffer containing 12 mM MgCl_2_ and a mixture of the four dNTPs (at 100 μM each) with or without 10 mg/ml sodium heparin. Aliquots of 4 μl were removed after 5, 15, 30 and 45 minutes and quenched with an equal volume of sample loading buffer. Products were resolved by denaturing polyacrylamide gel electrophoresis and analyzed as described above.

### RNase H assays

RNase H activity of mutant and WT RTs was determined with template-primers 31Trna/21P and D38Trna/25PGA as previously described [36,74].

## Competing interests

The authors declare that they have no competing interests.

## Authors’ contributions

GB obtained and characterized the recombinant RTs. CG carried out the analysis of the associations with resistance. MCP and MN, under the supervision of MAM and JMP performed experiments with recombinant viruses. LA, JLB, MJPE and CDM contributed reagents/materials/analysis tools and were responsible for patient recruitment and sample collection. GB, CG and LMA conceived and designed experiments. LMA wrote the manuscript and GB, CG, MAM and JMP helped to draft and edit the text. All authors read and approved the final version of the manuscript.

## Supplementary Material

Additional file 1 **Contains Tables S1, S2 and S3 and Figures S1 and S2.** Table S1. Correlated pairs of RT mutations in isolates from patients failing therapy with tenofovir and emtricitabine. Table S2. Factor analysis of the 25 correlated amino acid substitutions associated with tenofovir/emtricitabine therapy failure. Table S3. Dissociation equilibrium constants for WT and mutant HIV-1 RTs and DNA/DNA template-primers. Figure S1. Extension of unblocked DNA primer 25PGA by WT and mutant RTs in the presence of a DNA template. Reactions were carried out with template-primer concentrations of 1.5, 5 and 10 nM, in the presence of 3 nM RT and 100 μM of each dNTP. The represented values shown in the plots below were averages ± standard deviations [error bars], obtained from three independent experiments. Figure S2. Processivity of wild-type and mutant RTs. (A) Processivity assays with M13mp2 single-stranded DNA as template. Elongation reactions were monitored in the presence of heparin (5 mg/ml) as an enzyme trap. After formation of the binary complex of RT and template-primer (M13mp2 single-stranded DNA/ProLac110), reactions were initiated after addition of a mixture of all four dNTPs (50 μM final concentration), with or without heparin (indicated above with plus and minus signs, respectively). Lanes 1 to 4 represent samples taken 5, 15, 30 and 45 minutes after initiating the polymerization reaction. P stands for primer, and C represents control reactions where the enzyme was added after the heparin trap. The oligonucleotide ProLac110 (5’- GCGATTAAGTTGGGT-3’) is complementary to positions 105–119 of the *lacZα* coding sequence. (B) Processivity assays with M41L/L210W/T215Y and M41L/L210W/T215Y/R284K RTs using the heteropolymeric template-primer D38/25PGA. Assays were carried out in the same conditions described above for M13mp2 single-stranded DNA/ProLac110. (C) Relative amounts of extended primer in reactions carried out with D38/25PGA in the presence of trap. Asterisks indicate bands that are significantly more intense in the reactions catalyzed by the M41L/L210W/T215Y/R284K RT. Click here for file
