# Supplementary material for: Clinical, virological and biochemical evidence supporting the association of HIV-1 reverse transcriptase polymorphism R284K and thymidine analogue resistance mutations M41L, L210W and T215Y in patients failing tenofovir/emtricitabine therapy
Source: Retrovirology. 2012 Aug 13;9:68. doi: 10.1186/1742-4690-9-68 (PMC3468358; doi:10.1186/1742-4690-9-68)
Supplement: Additional file 1 — Contains Tables S1, S2 and S3 and Figures S1 and S2. Table S1. Correlated pairs of RT mutations in isolates from patients failing therapy with tenofovir and emtricitabine. Table S2. Factor analysis of the 25 correlated amino acid substitutions associated with tenofovir/emtricitabine therapy failure. Table S3. Dissociation equilibrium constants for WT and mutant HIV-1 RTs and DNA/DNA template-primers. Figure S1. Extension of unblocked DNA primer 25PGA by WT and mutant RTs in the presence of a DNA template. Reactions were carried out with template-primer concentrations of 1.5, 5 and 10 nM, in the presence of 3 nM RT and 100 μM of each dNTP. The represented values shown in the plots below were averages ± standard deviations [error bars], obtained from three independent experiments. Figure S2. Processivity of wild-type and mutant RTs. (A) Processivity assays with M13mp2 single-stranded DNA as template. Elongation reactions were monitored in the presence of heparin (5 mg/ml) as an enzyme trap. After formation of the binary complex of RT and template-primer (M13mp2 single-stranded DNA/ProLac110), reactions were initiated after addition of a mixture of all four dNTPs (50 μM final concentration), with or without heparin (indicated above with plus and minus signs, respectively). Lanes 1 to 4 represent samples taken 5, 15, 30 and 45 minutes after initiating the polymerization reaction. P stands for primer, and C represents control reactions where the enzyme was added after the heparin trap. The oligonucleotide ProLac110 (5’- GCGATTAAGTTGGGT-3’) is complementary to positions 105–119 of the lacZα coding sequence. (B) Processivity assays with M41L/L210W/T215Y and M41L/L210W/T215Y/R284K RTs using the heteropolymeric template-primer D38/25PGA. Assays were carried out in the same conditions described above for M13mp2 single-stranded DNA/ProLac110. (C) Relative amounts of extended primer in reactions carried out with D38/25PGA in the presence of trap. Asterisks indicate bands that a [file 1742-4690-9-68-S1.pdf]

Table S1. Correlated pairs of RT mutations in isolates from patients failing therapy with tenofovir and emtricitabine.[a]

Mutations		Phi	*P*-value[b]	
M41L	T215Y	0.89	1.04×10^{-19}	*
L210W	T215Y	0.78	1.87×10^{-15}	*
T69N	K219Q	0.76	7.39×10^{-15}	*
Q174R	L228H	0.76	1.02×10^{-14}	*
M41L	L210W	0.75	3.00×10^{-14}	*
A98G	Q174R	0.70	7.90×10^{-13}	*
T215F	L228H	0.63	1.30×10^{-10}	*
K70R	K219Q	0.61	5.34×10^{-10}	*
V118I	T215Y	0.59	1.39×10^{-9}	*
M184V	T215Y	0.57	6.21×10^{-9}	*
Y181C	K219E	0.54	4.70×10^{-8}	*
D67N	K70R	0.52	8.84×10^{-8}	*
T215F	K219Q	0.52	9.02×10^{-8}	*
M41L	V118I	0.52	1.17×10^{-7}	*
A98G	L228H	0.52	1.28×10^{-7}	*
M41L	M184V	0.50	3.23×10^{-7}	*
T69N	K70R	0.50	3.85×10^{-7}	*
K70R	K219E	0.50	3.85×10^{-7}	*
K70R	T215F	0.48	8.97×10^{-7}	*
M41L	V179I	0.48	1.01×10^{-6}	*
L74I	L228H	0.48	1.04×10^{-6}	*
A98G	G190A	0.48	1.15×10^{-6}	*
D67N	K219Q	0.47	1.65×10^{-6}	*
Q174R	G190A	0.47	2.01×10^{-6}	*
L74I	V179I	0.45	4.18×10^{-6}	*
Q174R	T215F	0.45	4.18×10^{-6}	*
T215F	K223E	0.65	5.02×10^{-6} [c]	*
V108I	H208Y	0.44	5.75×10^{-6}	*
A98G	Y181C	0.44	7.14×10^{-6}	*
A62V	Q174R	0.43	1.31×10^{-5}	*
T215Y	R284K	0.42	1.83×10^{-5}	
V108I	Y181C	0.42	2.28×10^{-5}	
V108I	L228R	0.41	2.34×10^{-5}	
V118I	V179I	0.41	2.42×10^{-5}	
V108I	M184V	0.40	3.87×10^{-5}	
V118I	M184V	0.40	3.87×10^{-5}	
M184V	L210W	0.40	4.33×10^{-5}	
I178L	H208Y	0.61	4.57×10^{-5} [c]	
T69N	L228H	0.40	5.51×10^{-5}	
A98G	H208Y	0.40	5.51×10^{-5}	

[a] Correlated pairs of mutations were determined from the 104 RT sequences obtained from patients receiving tenofovir and emtricitabine as the only RT inhibitors in the current antiretroviral regimen, and showing a viral load above 1000 RNA copies/ml. Data shown are the correlated pairs of mutations with phi values > 0.4. The *P*-values for the corresponding chi-squared contingency tests are given in the last column. Represented pairs are those with $P < 0.05$, using a Bonferroni correction. Asterisks indicate those pairs with high statistical significance ($P < 0.01$) by using the Bonferroni correction.

[b] *P* values have not been corrected for multiple comparisons.

[c] The significance of the correlation was determined with a Fisher's exact test, since I178L and K223E appeared only 4 and 5 times, respectively in the data set.

Table S2. Factor analysis of the 25 correlated amino acid substitutions associated with tenofovir/emtricitabine therapy failure.[a]

Amino acid substitution	Factor				
	1	2	3	4	5
M41L	0.031	0.908	-0.038	0.084	0.094
A62V	0.529	0.059	-0.062	0.021	0.184
D67N	-0.124	0.389	0.555	0.178	0.135
T69N	0.021	-0.076	0.735	-0.170	-0.096
K70R	-0.078	0.037	0.733	0.426	0.054
L74I	0.232	0.283	0.445	0.224	-0.314
A98G	0.744	0.148	-0.058	0.133	0.055
V108I	0.351	0.154	-0.019	-0.038	0.596
V118I	0.164	0.660	0.112	-0.032	0.062
Q174R	0.909	0.017	0.069	0.123	-0.110
I178L	0.726	-0.077	-0.028	-0.163	0.457
V179I	0.143	0.490	0.038	0.337	-0.187
Y181C	0.465	-0.009	0.071	0.534	0.277
M184V	0.352	0.506	0.176	0.078	0.191
G190A	0.487	0.055	0.046	0.089	-0.048
H208Y	0.492	0.205	0.102	-0.214	0.437
L210W	0.073	0.806	-0.034	0.088	0.108
T215F	0.422	-0.185	0.665	0.120	0.230
T215Y	0.060	0.939	-0.087	0.058	0.155
K219E	0.098	0.149	0.074	0.896	0.018
K219Q	-0.027	-0.048	0.884	-0.170	-0.119
K223E	0.155	-0.148	0.495	0.261	0.257
L228H	0.734	0.052	0.446	0.037	-0.114
L228R	-0.109	0.159	0.032	0.309	0.620
R284K	-0.082	0.356	-0.094	-0.053	-0.047

[a] Rotated factor matrix showing the three major associations contributing to therapy failure. The extraction method employed was principal axis factoring, continued of rotation by the Varimax with Kaiser normalization method. Rotation converged in ten iterations. Numbers in red, blue and purple are used to highlight values above 0.6, between 0.4 and 0.6, and between 0.3 and 0.4, respectively.

Table S3. Dissociation equilibrium constants for WT and mutant HIV-1 RTs and DNA/DNA template-primers.

RTs	Apparent K_d (nM)
WT	1.65 ± 0.48
R284K	1.94 ± 0.42
M41L/L210W/T215Y	2.37 ± 0.65
M41L/L210W/T215Y/R284K	1.71 ± 0.21

The K_d values for DNA/DNA binding were obtained with the template-primer D38/25PGA. Reported values are the averages ± standard deviations, obtained from at least three independent experiments.

.

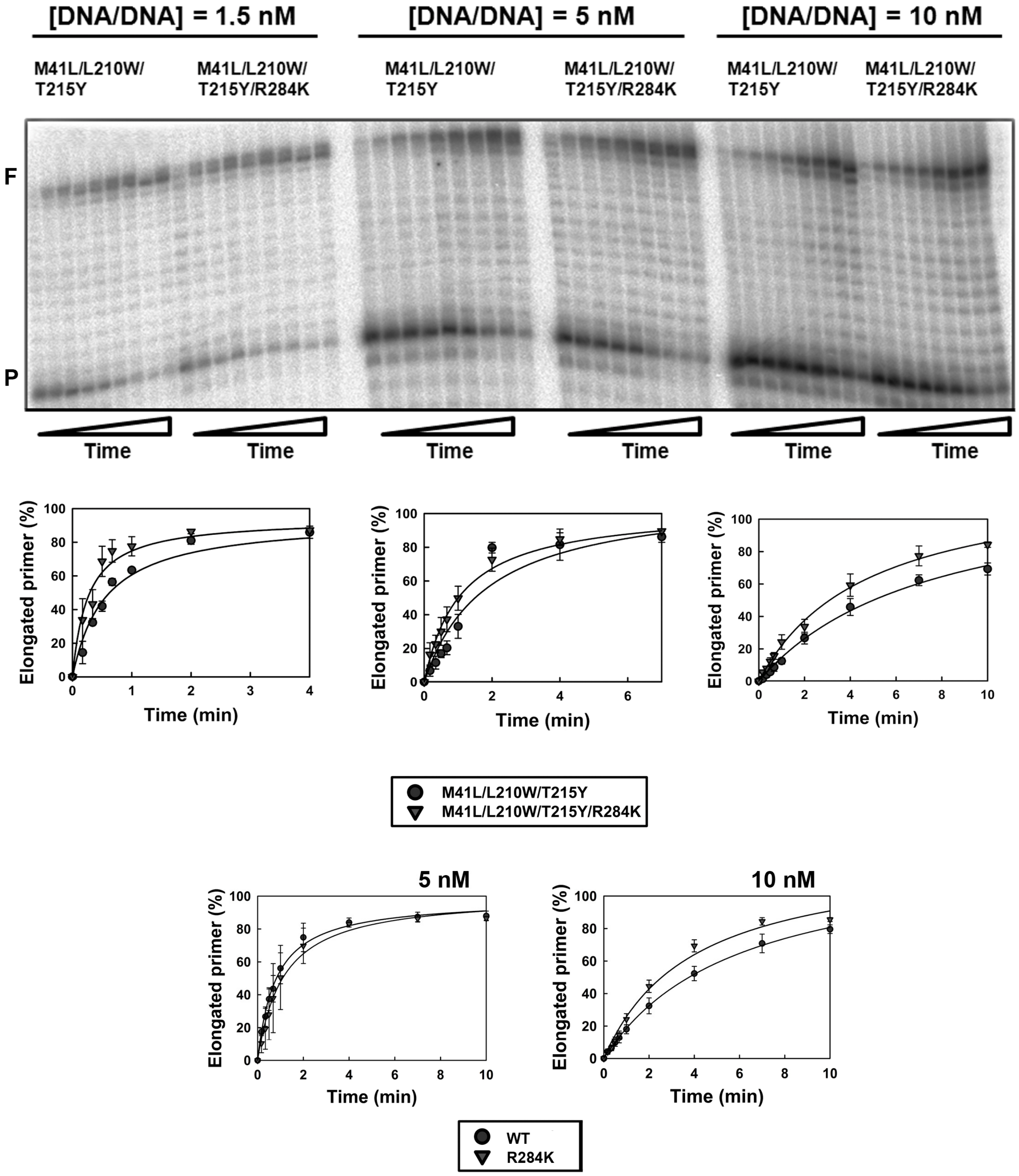

Figure S1. Extension of unblocked DNA primer 25PGA by WT and mutant RTs in the presence of a DNA template. Reactions were carried out with template-primer concentrations of 1.5, 5 and 10 nM, in the presence of 3 nM RT and 100 µM of each dNTP. The represented values shown in the plots below were averages ± standard deviations [error bars], obtained from three independent experiments.

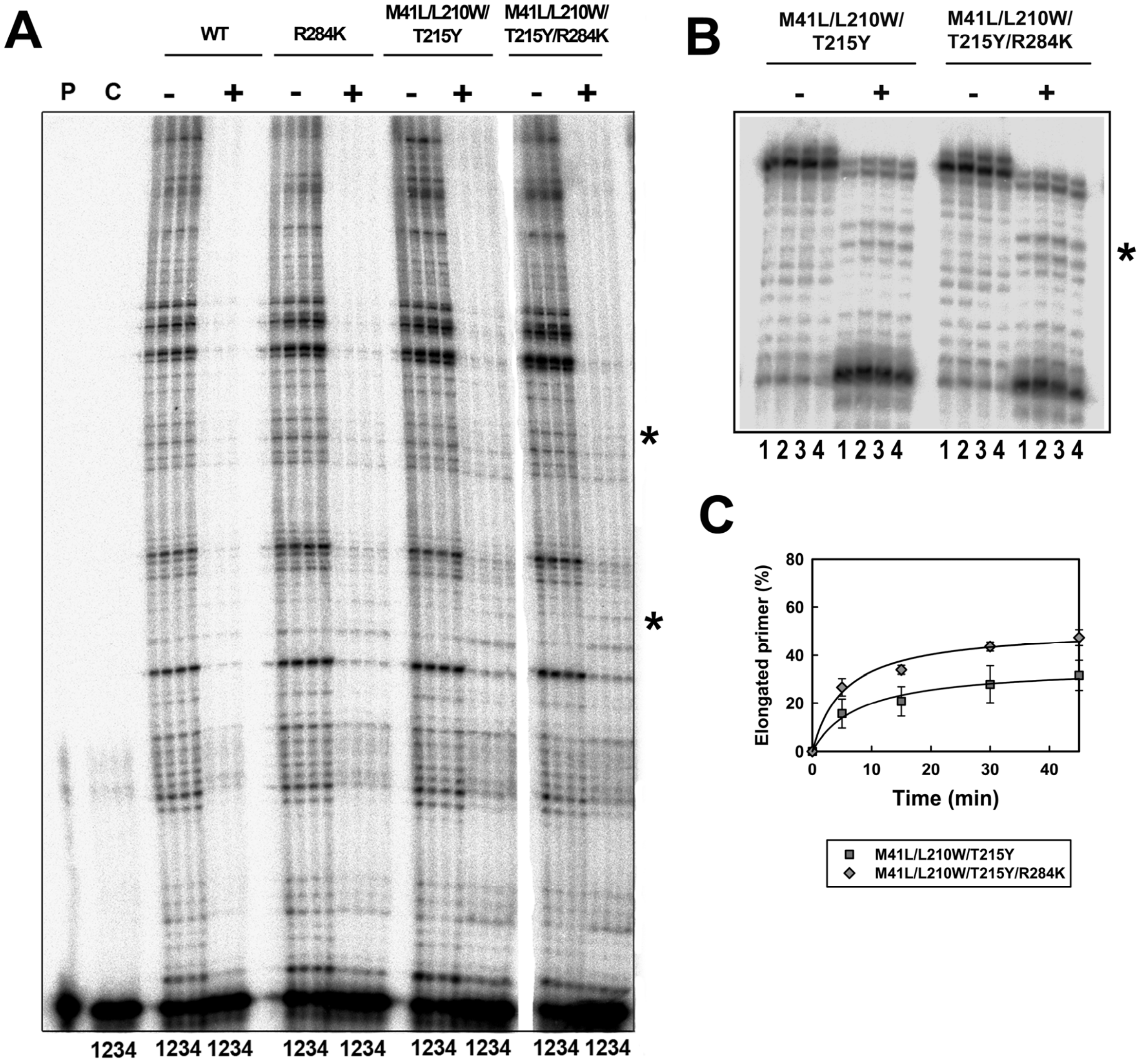

Figure S2. Processivity of wild-type and mutant RTs. (A) Processivity assays with M13mp2 single-stranded DNA as template. Elongation reactions were monitored in the presence of heparin (5 mg/ml) as an enzyme trap. After formation of the binary complex of RT and template-primer (M13mp2 single-stranded DNA/ProLac110), reactions were initiated after addition of a mixture of all four dNTPs (50 μM final concentration), with or without heparin (indicated above with plus and minus signs, respectively). Lanes 1 to 4 represent samples taken 5, 15, 30 and 45 min after initiating the polymerization reaction. P stands for primer, and C represents control reactions where the enzyme was added after the heparin trap. The oligonucleotide ProLac110 (5'- GCGATTAAGTTGGGT-3') is complementary to positions 105-119 of the *lacZα* coding sequence. (B) Processivity assays with M41L/L210W/T215Y and M41L/L210W/T215Y/R284K RTs using the heteropolymeric template-primer D38/25PGA. Assays were carried out in the same conditions described above for M13mp2 single-stranded DNA/ProLac110. (C) Relative amounts of extended primer in reactions carried out with D38/25PGA in the presence of trap. Asterisks indicate bands that are significantly more intense in the reactions catalyzed by the M41L/L210W/T215Y/R284K RT.
